# Necrotizing pneumonia due to methicillin-sensitive *Staphylococcus aureus *secreting Panton-Valentine leukocidin: a review of case reports

**DOI:** 10.1186/cc10649

**Published:** 2012-03-20

**Authors:** L Kreienbuehl, E Charbonney, P Eggimann

**Affiliations:** 1 HUG, Geneva, Switzerland; 2 Li Ka Shing Knowledge Institute, St Michael's Hospital, Toronto, Canada; 3 CHUV, Lausanne, Switzerland

## Introduction

Community-acquired necrotizing pneumonia caused by Panton-Valentine leukocidin (PVL)-secreting *Staphylococcus aureus *is a highly lethal infection, which mainly affects healthy children and young adults [1,2]. This study focuses on necrotizing pneumonia due to methicillin-sensitive *S. aureus *strains, with the purpose to determine factors associated with outcome.

## Methods

We performed a systematic review of case reports on PVL-secreting MSSA necrotizing pneumonia and analyzed factors associated with outcome.

## Results

A total of 32 patient descriptions were retained for analysis. Septic shock, influenza-like prodrome and the absence of a previous skin and soft tissue infection were associated with fatal outcome. In multivariate analysis, influenza-like prodrome (OR 7.44; 95% CI: 1.24 to 44.76; *P *= 0.028) and absence of previous skin and soft tissue infection (OR 0.09; 95% CI: 0.010 to 0.86; *P *= 0.036) remained significant predictors of death. See Table 1.

**Table 1:** Univariate analysis of mortality risk factors

	Died (*n *= 13)	Survived (*n *= 19)	Univariate analysis OR (95% CI)	*P *value
Flu-like prodrome	9/12 (75%)	4/16 (25%)	9.00 (1.60 to 50.7)	0.020
SSTI	1/13 (8%)	9/19 (47%)	0.09 (0.01 to 0.86)	0.024
Septic shock	11/11	7/15 (47%)	26.0 (1.30 to 522)	0.007
Leukocytopenia	9/11 (82%)	8/17 (47%)	5.06 (0.83 to 30.8)	0.115

## Conclusion

Influenza-like prodrome may be predictive of adverse outcome and previous skin and soft tissue infection may be associated with improved prognosis.
